# Functional cytochrome P450 1A enzymes are induced in mouse and human islets following pollutant exposure

**DOI:** 10.1007/s00125-019-05035-0

**Published:** 2019-11-27

**Authors:** Muna Ibrahim, Erin M. MacFarlane, Geronimo Matteo, Myriam P. Hoyeck, Kayleigh R. C. Rick, Salar Farokhi, Catherine M. Copley, Shannon O’Dwyer, Jennifer E. Bruin

**Affiliations:** 1 Laboratory of Molecular and Cellular Medicine, Department of Cellular & Physiological Sciences, Life Sciences Institute, University of British Columbia, Vancouver, BC Canada; 2 Department of Biology, Carleton University, 1125 Colonel By Drive, Ottawa, ON K1S 5B6 Canada; 3 Institute of Biochemistry, Carleton University, 1125 Colonel By Drive, Ottawa, ON K1S 5B6 Canada

**Keywords:** Diabetes, Drug metabolism enzymes, Islets, Pollutants, Toxicology, Xenobiotics

## Abstract

**Aims/hypothesis:**

Exposure to environmental pollution has been consistently linked to diabetes incidence in humans, but the potential causative mechanisms remain unclear. Given the critical role of regulated insulin secretion in maintaining glucose homeostasis, environmental chemicals that reach the endocrine pancreas and cause beta cell injury are of particular concern. We propose that cytochrome P450 (CYP) enzymes, which are involved in metabolising xenobiotics, could serve as a useful biomarker for direct exposure of islets to pollutants. Moreover, functional CYP enzymes in islets could also impact beta cell physiology. The aim of this study was to determine whether CYP1A enzymes are activated in islets following direct or systemic exposure to environmental pollutants.

**Methods:**

Immortalised liver (HepG2) and rodent pancreatic endocrine cell lines (MIN6, βTC-6, INS1, α-TC1, α-TC3), as well as human islets, were treated in vitro with known CYP1A inducers 2,3,7,8-tetrachlorodibenzo*-p-*dioxin (TCDD) and 3-methylcholanthrene (3-MC)*.* In addition, mice were injected with either a single high dose of TCDD or multiple low doses of TCDD in vivo*,* and islets were isolated 1, 7 or 14 days later.

**Results:**

CYP1A enzymes were not activated in any of the immortalised beta or alpha cell lines tested. However, both 3-MC and TCDD potently induced *CYP1A1* gene expression and modestly increased CYP1A1 enzyme activity in human islets after 48 h. The induction of *CYP1A1* in human islets by TCDD was prevented by cotreatment with a cytokine mixture. After a systemic single high-dose TCDD injection, CYP1A1 enzyme activity was induced in mouse islets ~2-fold, ~40-fold and ~80-fold compared with controls after 1, 7 and 14 days, respectively, in vivo. Multiple low-dose TCDD exposure in vivo also caused significant upregulation of *Cyp1a1* in mouse islets. Direct TCDD exposure to human and mouse islets in vitro resulted in suppressed glucose-induced insulin secretion. A single high-dose TCDD injection resulted in lower plasma insulin levels, as well as a pronounced increase in beta cell death.

**Conclusions/interpretation:**

Transient exposure to TCDD results in long-term upregulation of CYP1A1 enzyme activity in islets. This provides evidence for direct exposure of islets to lipophilic pollutants in vivo and may have implications for islet physiology.

**Electronic supplementary material:**

The online version of this article (10.1007/s00125-019-05035-0) contains peer-reviewed but unedited supplementary material, which is available to authorised users.

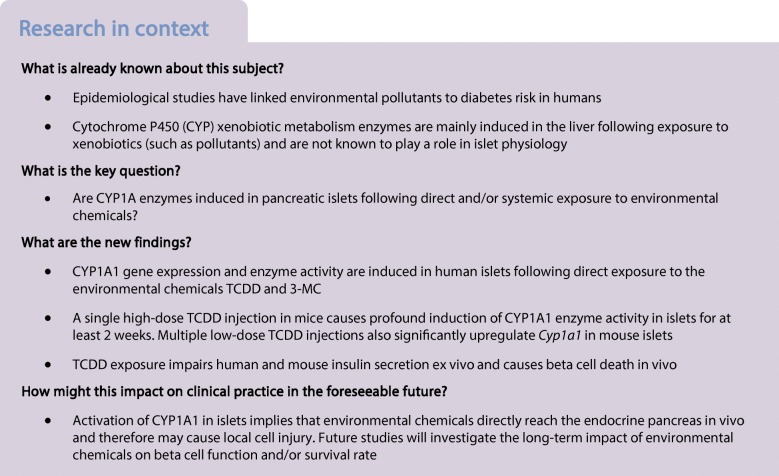



## Introduction

Persistent organic pollutants (POPs) are lipophilic and resistant to degradation, resulting in widespread environmental dispersion and bioaccumulation [1, 2]. Chronic human exposure to POPs is associated with adverse health outcomes [3–8], including increased diabetes risk [9–25] and clinical measures of poor insulin secretion [26–28]. However, the mechanisms underlying these associations remain poorly understood.

The pancreas is not traditionally considered a target organ for environmental chemicals, yet mounting evidence suggests that pollutants, particularly POPs, may impact beta cell function [26, 29–31]. For example, reduced insulin secretion was observed in isolated islets 24 h after in vivo exposure to dioxin (2,3,7,8-tetrachlorodibenzo*-p-*dioxin; TCDD) [30, 31]. Dioxin/dioxin-like compounds are a broad class of POPs that act as agonists for the aryl hydrocarbon receptor (AhR), which activates AhR target genes, including *CYP1A1* and *CYP1A2* [32, 33]. The primary role for cytochrome P450 (CYP) enzymes is xenobiotic metabolism and detoxification, but the reactive metabolites generated by CYP-mediated oxidation can be highly toxic. These metabolites are generally unstable and act locally to cause oxidative stress and DNA/protein damage [32–34]. Although CYP enzymes mainly act in the liver, non-hepatic CYP enzymes have also been described [35, 36]. Interestingly, non-hepatic tissues typically accumulate substantially lower concentrations of xenobiotics than liver, but can be disproportionately sensitive to their effects. For example, following TCDD administration in mice, TCDD concentrations were 100 times higher in liver than lung, but CYP1A1 activity was two times higher in lung than liver [37]. We hypothesised that CYP enzymes would be inducible in the endocrine pancreas and serve as a useful tool to elucidate whether environmental chemicals directly target islet cells in vivo.

Previous data supported our idea that CYP1A enzymes might be inducible in the endocrine pancreas. A modest increase in CYP1A1 protein was detected by western blot in immortalised MIN-6 beta cells following 24 h TCDD treatment [38]. ‘CYP1A-like’ proteins were reportedly induced in pancreas sections from rats following in vivo 3-methylcholanthrene (3-MC) exposure, but these data relied on a promiscuous polyclonal antibody [39]. Most notably, O-dealkylation of 7-ethoxyrosorufin (EROD), an established assay for CYP1A1 activity, was increased in pancreatic microsomes from 3-MC-injected rats compared with controls [40]. It remains unclear whether CYP1A1/1A2 are upregulated and/or functional in islets, particularly human tissues. Here, we investigated whether *CYP1A* genes are induced in mouse and/or human islets following direct in vitro exposure to xenobiotics, TCDD and 3-MC, or systemic exposure in vivo. We also used enzyme activity assays to determine whether islets harbour functional CYP1A enzymes capable of substrate metabolism.

## Methods

### Cell culture

HepG2 cells (kindly provided by T. Kieffer, University of British Columbia), an immortalised human liver cell line, were cultured in high-glucose (25 mmol/l) DMEM (DMEM-HG; #10-013-CV, Corning, Corning, NY, USA; or #D6429, Sigma-Aldrich, St Louis, MO, USA) with 10% (vol./vol.) heat-inactivated FBS (Sigma-Aldrich #F1051). INS-1 cells (kindly provided by C. Wollheim, University Medical Center, Geneva, Switzerland), an immortalised rat beta cell line, were cultured in RPMI 1640 (Corning #10-041-CV) with 10% (vol./vol.) FBS, 50 μmol/l 2-mercaptoethanol (Sigma-Aldrich), 10 mmol/l HEPES (#BP310, Thermo Fisher Scientific, Waltham, MA, USA) and 1 mmol/l sodium pyruvate (Sigma-Aldrich #S8636). MIN6 cells (kindly provided by J. Miyazaki, Osaka University Graduate School of Medicine, Osaka, Japan), an immortalised mouse beta cell line, were cultured in DMEM-HG with 10% (vol./vol.) FBS. βTC-6 cells (#CRL-11506, ATCC, Manassas, VA, USA), an immortalised mouse beta cell line, were cultured in DMEM-HG with 15% (vol./vol.) FBS. α-TC3 and α-TC1 clone nine cells (kindly provided by T. Kieffer), immortalised mouse alpha cell lines, were cultured in DMEM-HG (Sigma-Aldrich #D6429A) with 10% (vol./vol.) FBS. All cell lines were confirmed to be free of mycoplasma using the MycoAlert Mycoplasma Detection Kit (#CA11006554; Lonza, Basel, Switzerland).

### Mouse islet isolation and culture

Islets were isolated from mice by pancreatic duct injection with collagenase (1000 U/ml; Sigma-Aldrich #C7657) dissolved in Hanks’ balanced salt solution (HBSS: 137 mmol/l NaCl, 5.4 mmol/l KCl, 4.2 mmol/l NaH_2_PO_4_, 4.1 mmol/l KH_2_PO_4_, 10 mmol/l HEPES, 1 mmol/l MgCl_2_, 5 mmol/l dextrose, pH 7.2). Pancreases were incubated at 37°C for 12 min, vigorously agitated and the collagenase reaction quenched by adding cold HBSS with 1 mmol/l CaCl_2_. The pancreas tissue was washed three times in HBSS+CaCl_2_ (centrifuging for 1 min at 1000 *g* in between washes) and resuspended in Ham’s F-10 (#SH30025.01, HyClone, GE Healthcare Bio-sciences, Pittsburgh, PA, USA; or Corning #10-070-CV) containing 0.5% (wt/vol.) BSA (Sigma-Aldrich #10775835001), 100 U/ml penicillin and 100 μg/ml streptomycin (Corning #30002CI). Pancreas tissue was filtered through a 70 μm cell strainer and islets were handpicked under a dissecting scope to >95% purity.

### Human islet culture

Human islets for experiments in Figs. 3a, b, d, e (H216), 3c (H219), 3f–h (H210, H211, H220) and 4d (H215) were obtained from the Ike Barber Human Islet Transplant Laboratory (Vancouver, BC, Canada). Organ donor purity ranged from 60% to 90% islets. Human islets for experiments in Fig. 4a–c (R161) were obtained from the Alberta Diabetes Institute IsletCore (Edmonton, AB, Canada) and islet purity was 95%. All human islets were cultured in CMRL medium (Gibco #11530-037; Thermo Fisher Scientific) with 10% (vol/vol) FBS, 100 U/ml penicillin, 100 μg/ml streptomycin and 2 mmol/l l-glutamine (Sigma-Aldrich #59202C). Research with human islets was approved by the Research Ethics Boards at the University of British Columbia and Carleton University. Refer to the electronic supplementary material (ESM) for the Human Islets Checklist.

### In vitro cell treatments

To determine whether CYP1A1 could be induced in islets, we selected doses that are well established to maximally induce CYP1A1 in liver cells without causing toxicity. The upper doses reflect the half maximal inhibitory concentration (EC_50_) for each chemical. Since TCDD is 1000 times more potent than 3-MC [41–43], we used lower doses of TCDD (1–10 nmol/l) compared with 3-MC (100–1000 nmol/l). Adherent cell lines and isolated pancreatic islets (mouse and human) were treated for 48 h in vitro with 0.1 μmol/l or 1.0 μmol/l 3-MC (Sigma-Aldrich #46434-2ML-R, 100 ng/μl solution in acetonitrile), 1 nmol/l or 10 nmol/l TCDD (Sigma-Aldrich #48599, 10 μg/ml solution in toluene) or the appropriate vehicle control (acetonitrile, toluene or DMSO) in culture medium. Medium was refreshed after 24 h. Where indicated, human islets were also treated with 1 μmol/l thapsigargin (Sigma-Aldrich), a cytokine mixture (TNF-α, 50 ng/ml, #510-RT-010, R&D Systems, Minneapolis, MN, USA; IFNγ, 1000 U/ml, #213-10156-1AF, RayBiotech, Peachtree Corners, GA, USA; IL-1β, 10 ng/ml, #JM-4128-10, MBL International Corporation, Woburn, MA, USA) or 10 nmol/l exendin-4 (Sigma-Aldrich #E7144)

### CYP enzyme activity assay

Enzyme activity was measured in various adherent cell lines (HepG2, MIN6, βTC-6, INS1, α-TC1, α-TC3) and isolated mouse and human islets using the P450-Glo CYP1A1 Assay (#V8752; Promega, Madison, WI, USA) and CYP1A2 Assay (#V8772, Promega). All assays were performed in 96-well white-walled plates with clear bottoms (#655098; Greiner Bio-One, Kremsmünster, Austria) using the lytic method, as described by the manufacturer. For adherent cell lines, the assay was performed on cells at 70–90% confluence. For islets, 50 mouse or human islets were handpicked into each well of the 96-well plate.

### In vitro glucose-stimulated insulin secretion assays

To assess beta cell function, 25–50 mouse or human islets per condition were transferred to a pre-warmed (37°C) 24-well plate containing Krebs–Ringer bicarbonate buffer (KRBB) (115 mmol/l NaCl, 5 mmol/l KCl, 24 mmol/l NaHCO_3_, 2.5 mmol/l CaCl_2_, 1 mmol/l MgCl_2_, 10 mmol/l HEPES, 0.1% (wt/vol.) BSA, pH 7.4) with 2.9 mmol/l glucose (low glucose, LG) for a 40 min pre-incubation at 37°C. Islets were then transferred to 500 μl of LG KRBB for 1 h, followed by transfer to 500 μl of KRBB with 16.7 mmol/l glucose (high glucose, HG) for 1 h at 37°C. The LG KRBB and HG KRBB samples were centrifuged, and the supernatant stored at −30°C until use. To measure insulin content, islets were transferred to an acid-ethanol solution of 1.5% (vol./vol.) HCl in 70% (vol./vol.) ethanol at 4°C overnight and then neutralised with 1 mol/l Tris base (pH 7.5) before long-term storage at −30°C. Concentrations of human C-peptide (#10-1141-01; Mercodia, Uppsala, Sweden) and mouse insulin (#80-INSMS or 80-INSMSH; ALPCO, Salem, NH, USA) were measured by ELISA.

### Animals

All mice received ad libitum access to a standard irradiated diet (Teklad Diet #2918; Harlan Laboratories, Madison, WI, USA) and were maintained on a 12 h light/dark cycle throughout the study. All experiments were approved by the University of British Columbia or Carleton University Animal Care Committees and carried out in accordance with the Canadian Council on Animal Care guidelines. Prior to beginning experimental protocols, animals were randomly assigned to treatment groups and matched for body weight and blood glucose levels (ensuring that these variables were not significantly different between groups).

#### Cohort 1

As outlined in Fig. 5a, 8-week-old male C57Bl/6 mice (Jackson Laboratory, Bar Harbour, ME, USA) received a single i.p. injection of corn oil (25 ml/kg, vehicle control; *n* = 4), 20 μg/kg TCDD (*n* = 4), 100 μg/kg TCDD (*n* = 4) or 200 μg/kg TCDD (*n* = 4). Liver was flash frozen in liquid nitrogen and islets were isolated from all mice (as described above) 24 h after injection.

#### Cohort 2

As outlined in Figs. 5h, 6a, 8-week-old male C57Bl/6 mice (Jackson Laboratory) received a single i.p. injection of corn oil (25 ml/kg, vehicle control; *n* = 24) or 200 μg/kg TCDD (*n* = 22). On days 7 (*n* = 9–10 per group) and 14 (*n* = 9–11 per group) following injection, islets were isolated from a subset of mice for ex vivo glucose-stimulated insulin secretion assays (25 islets per mouse), RNA isolation (~150–200 islets per mouse) and CYP1A enzyme activity assays (50 islets per mouse per assay). Liver was flash frozen in liquid nitrogen on days 7 (*n* = 5 per group) and 14 (*n* = 9–11 per group). Whole pancreas and liver were harvested from a different subset of mice on day 7 (*n* = 3 per group) and stored in 4% (vol./vol.) paraformaldehyde (PFA) for 24 h, followed by long-term storage in 70% (vol./vol.) ethanol.

#### Cohort 3

As outlined in Fig. 5m and ESM Fig. 2a, 8-week-old male C57Bl/6 mice received multiple i.p. injections of corn oil (25 ml/kg, vehicle control) or a low-dose of TCDD (20 ng/kg per day) twice per week. This dose was previously shown to induce CYP1A1 in liver and lung [44, 45]. Furthermore, chronic administration of 46 ng/kg TCDD per day resulted in circulating TCDD concentrations of ~8.8 pg/g in rats [44], which is within the range of background dioxin levels reported in the USA (≤10 pg/g) and corresponds to individuals in the upper quartile (≥5.2 pg/g) with increased diabetes prevalence [16]. In our study, the first group of mice (Fig. 5m–p; Jackson Laboratory) was treated at the University of Ottawa Roger Guindon Hall vivarium (*n* = 5 control, *n* = 8 TCDD); liver was flash frozen and islets were isolated after 2 weeks (i.e. five injections). A second group of mice (ESM Fig. 2) was generated by in-house breeding and then treated in the Modified Barrier Facility at the University of British Columbia (*n* = 4 per group) for metabolic assessment.

#### *Cyp1a1*/*1a2* double knockout mice

Mice with a global deletion of both *Cyp1a1* and *Cyp1a2*, originally generated and characterised by D. Nebert [46], were generously provided by F. Gonzalez (University of Cincinnati). Male mice were used for ex vivo experiments with isolated islets (Fig. 7) and as a negative control for CYP1A1 immunofluorescence staining of pancreas and liver tissues (ESM Fig. 1).

### Metabolic assessments

All metabolic analyses were performed in conscious, restrained mice and blood samples were collected via saphenous vein using heparinised microhematocrit tubes at the indicated time points. Blood glucose levels were measured using a handheld glucometer (Lifescan, Burnaby, BC, Canada).

Body weight and blood glucose levels were assessed weekly or bi-weekly following a 4 h morning fast. For all other metabolic tests, time 0 indicates the blood sample collected after fasting and prior to administration of glucose or insulin. For GTTs, mice received an i.p. bolus of glucose (2 g/kg; Vetoquinol, Lavaltrie, QC, Canada) following a 6 h morning fast. During the GTT, blood was collected at the indicated time points for measuring plasma insulin levels by ELISA (ALPCO mouse ultrasensitive insulin ELISA, #80-INSMSU-E01). For ITTs, mice received an i.p. bolus of insulin (0.7 U/kg, Novolin ge Toronto #02024233; Novo Nordisk Canada, Mississauga, ON, Canada) after a 4 h morning fast. For all tests, mice from different treatment groups were randomly distributed throughout the experiment to ensure that timing of blood collection was not a factor in our analysis.

### Quantitative real-time PCR

RNA was isolated from cultured cell lines using the RNeasy Mini Kit (#74104; Qiagen, Hilden, Germany), human or mouse islets using the RNeasy Micro Kit (#74004; Qiagen) and liver using TRIzol reagent (#15596018; Invitrogen, Carlsbad, CA, USA) or the Qiagen RNeasy Mini Kit, according to the manufacturer instructions. DNase treatment was performed prior to cDNA synthesis with the iScript gDNA Clear cDNA Synthesis Kit (#1725035; Bio-Rad, Mississauga, ON, Canada). Quantitative real-time PCR (qPCR) was performed using the SsoFast EvaGreen Supermix (#1725200; Bio-Rad) or SsoAdvanced Universal SYBR Green Supermix (#1725271, Bio-Rad) and run on a CFX96 or CFX384 (Bio-Rad). *Hprt*/*HPRT* or *Ppia*/*PPIA* were used as the reference genes. Data were analysed using the ΔΔC_t_ method. Primer sequences are listed in ESM Table 1.

### Immunofluorescent staining and image quantification

Human islets were washed with PBS, resuspended in 4% (wt/vol.) PFA and stored overnight at 4°C. The following day, islets were washed twice with PBS, resuspended in 200 μl pre-warmed 2% (wt/vol.) agarose and cooled briefly at −20°C. The agarose pellet was resuspended in 4% (wt/vol.) PFA for 1 h at room temperature and then transferred to 70% (vol/vol.) ethanol for long-term storage at 4°C until paraffin embedding. Whole pancreas tissue was fixed in 4% (vol./vol.) PFA for 24 h and stored in 70% (vol./vol.) ethanol prior to paraffin embedding.

Paraffin sections (5 μm thickness) were prepared by Wax-it Histology Services (Vancouver, BC, Canada) or the University of Ottawa Heart Institute Histology Core Facility (Ottawa, ON, Canada). Briefly, slides were deparaffinised with sequential 5 min incubations in xylene (×3), 100% (vol./vol.) ethanol (×2), 95% (vol./vol.) ethanol (×1) and 70% (vol./vol.) ethanol (×1), and then transferred to PBS for 10 min on a shaker. Heat-induced epitope retrieval (HIER) was performed in citrate buffer (10 mmol/l sodium citrate, 0.05% (vol./vol.) Tween 20, pH 6.0) for 10 min at 95°C using the EZ Retriever microwave system (#MW014-MO; BioGenex, Fremont, CA, USA), unless indicated otherwise below. Slides were transferred to deionised water for a 5 min rinse and then to PBS for 5 min. Tissue sections were circumscribed with an ImmEdge Pen (#H-4000; Vector Laboratories, Burlingame, CA, USA) and then incubated at room temperature for a minimum of 30 min with Dako serum-free protein block (#X090930-2; Agilent, Santa Clara, CA, USA). Next, primary antibodies were diluted as indicated below for each antibody and added to each section; slides were stored in a humid chamber overnight at 4°C. The following day, slides were washed 3×10 min in PBS on a shaker and incubated in secondary antibodies for 1 h in a humid chamber at room temperature, protected from light. Finally, slides were washed 3×10 min in PBS on a shaker, and then coverslips were mounted with VECTASHIELD HardSet Mounting Medium with DAPI (#H-1500, Vector Laboratories) for counterstaining.

The following primary antibodies were used in this study: rabbit anti-MAFA (MAF bZIP transcription factor A) (15 min HIER, 1:1000, shared by T. Kieffer), rabbit anti-somatostatin (1:500, Sigma-Aldrich #HPA019472), rabbit anti-insulin (C27C9, 1:200, Cell Signaling Technology, Danvers, MA, USA; #3014), mouse anti-insulin (L6B10, 1:250, Cell Signaling Technology #8138BF) and mouse anti-glucagon (1:1000, Sigma-Aldrich #G2654). Numerous primary antibodies for CYP1A1 were tested, and these are outlined in ESM Table 2. The following secondary antibodies were used: goat anti-rabbit IgG (H+L) secondary antibody, Alexa Fluor 594 (1:1000, Invitrogen #A11037); and goat anti-mouse IgG (H+L) secondary antibody, Alexa Fluor 488 (1:1000, Invitrogen #A11029). All antibodies were diluted using Dako Background Reducing Antibody Diluent solution (#S302283-2, Agilent).

To measure apoptosis, pancreas and liver sections were stained with the Molecular Probes Click-iT Plus TUNEL Assay using Alexa Fluor 488 dye (Invitrogen #C10617), according to the manufacturers instructions. Pancreas sections were counterstained with rabbit anti-insulin (C27C9, 1:200, Cell Signaling #3014) and goat anti-rabbit IgG (H+L) secondary antibody, Alexa Fluor 594 (1:1000, Invitrogen #A11037). No antigen retrieval was used for counterstaining.

For islet morphology quantification, a minimum of eight islets were imaged with an EVOS FL Cell Imaging System (Invitrogen); the islet area, insulin-immuonoreactive area and glucagon-immunoreactive area were measured for each islet using ImageJ software (https://imagej.nih.gov/ij/index.html). Islet cell apoptosis was quantified as the percentage of insulin^+^ and insulin^−^ islet cells that colocalised with TUNEL^+^ nuclei. Liver apoptosis was quantified in at least five images per mouse and expressed as the percentage of TUNEL^+^ cells in the field of view. Images were captured and quantified by a researcher blinded to the treatment groups.

H&E staining of pancreas and liver tissues was performed using standard protocols by Wax-it Histology Services. Colour images were taken with an Axio Observer 7 microscope (ZEISS, Oberkochen, Germany).

### Statistical analysis

All statistics were performed using GraphPad Prism software (GraphPad Software, La Jolla, CA, USA). Specific statistical tests are indicated in the figure legends. For all analyses, *p* < 0.05 was considered statistically significant. Data are presented as mean ± SEM, unless indicated otherwise.

## Results

### TCDD levels in pancreas decline slower than other tissues in vivo

**Fig. 1:**
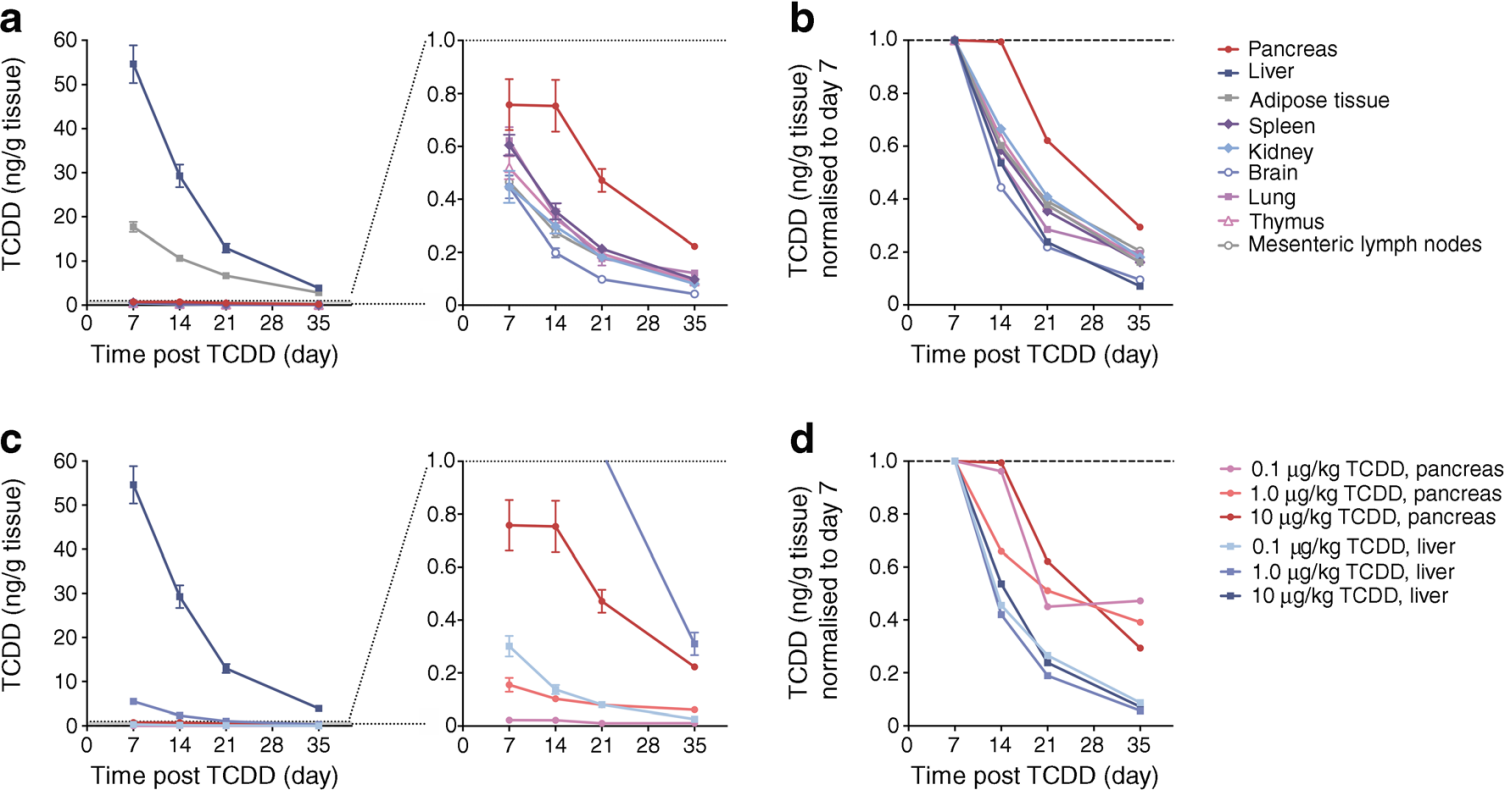
TCDD levels are more stable in the pancreas than in other tissues. Data from a published biodistribution study [37] were re-analysed to compare TCDD levels in the pancreas with other mouse tissues at 7, 14, 21 and 35 days following oral administration of TCDD (0.1, 1.0 or 10 μg/kg) to female B6C3F1 mice (*n* = 5 mice per group). (**a**, **b**) Concentration of TCDD (ng/g tissue) in various tissues after administration of 10 μg/kg TCDD. (**c**, **d**) Concentration of TCDD (ng/g tissue) in liver and pancreas after oral administration of 0.1, 1.0 or 10 μg/kg TCDD. Data are presented as mean ± SD (**a**, **c**) and mean TCDD concentration normalised to day 7 (**b**, **d**)

To determine how much TCDD reaches the pancreas, we used data from a biodistribution study that measured TCDD tissue concentrations after TCDD administration in mice [37]. After 7 days, the TCDD concentration in pancreas was 72 times lower than liver and 23 times lower than adipose, but higher than other non-classical target tissues (Fig. 1a). However, TCDD levels declined slower in the pancreas than all other tissues (Fig. 1b, d). In liver, TCDD levels were 72 times higher than pancreas 7 days after 10 μg/kg TCDD administration, but only 17 times higher after 35 days (Fig. 1c), and this gap diminished further at lower doses. On day 35, TCDD levels were only five times higher in liver than pancreas after a 1.0 μg/kg dose and 2.4 times higher after a 0.1 μg/kg dose (Fig. 1c). Between days 7 and 35 the concentration of TCDD declined by 91–94% in liver, compared with only 50–70% in pancreas (Fig. 1d). These data indicate that TCDD may be more stable in the pancreas than classical target tissues.

### CYP1A enzymes are not induced in immortalised beta or alpha cell lines

**Fig. 2:**
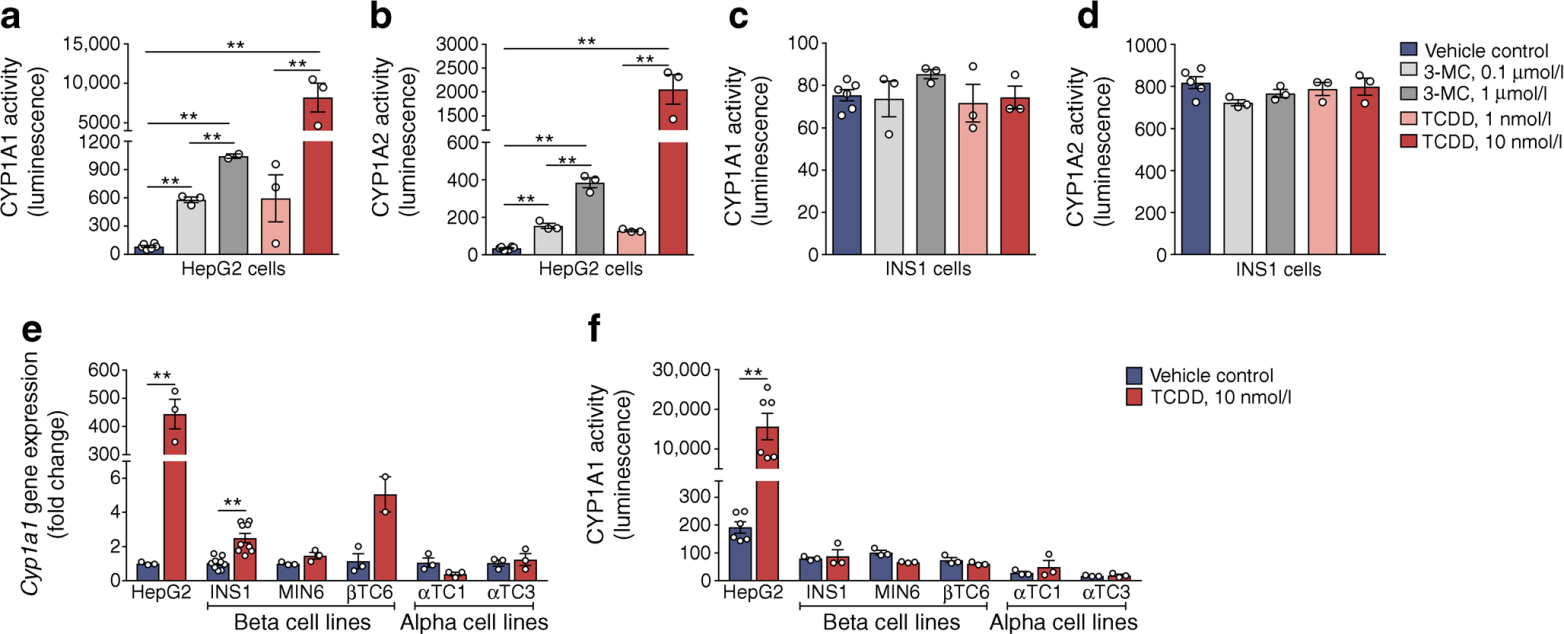
Xenobiotics do not induce CYP1A enzymes in immortalised pancreatic endocrine cell lines. Liver cells (HepG2), beta cell lines (INS1, MIN6, βTC6) and alpha cell lines (αTC1 and αTC3) were treated for 48 h with media containing vehicle (control), 3-MC (0.1 or 1.0 μmol/l) or TCDD (1.0 or 10 nmol/l). (**a**–**d**) Following treatment, CYP1A1 and CYP1A2 enzyme activity was measured in HepG2 (**a**, **b**) and INS1 cells (**c**, **d**). **p < 0.01, one-way ANOVA with Tukey’s post hoc test. (**e**) *Cyp1a1* gene expression and (**f**) CYP1A1 enzyme activity were measured after exposure to vehicle (DMSO) or 10 nmol/l TCDD in all immortalised cell lines. **p < 0.01, unpaired two-tailed *t* test. All data are presented as mean ± SEM and individual data points represent technical replicates of cells treated independently in different wells

To test our hypothesis that CYP1A enzymes are inducible in pancreatic endocrine cells, we first treated INS1 cells and HepG2 liver cells with CYP1A inducers TCDD and 3-MC. Both chemicals profoundly increased CYP1A1 and CYP1A2 enzyme activity in HepG2 cells (Fig. 2a, b), but not in INS1 cells (Fig. 2c, d). A modest but statistically significant ~2.4-fold increase in *Cyp1a1* gene expression was detected in TCDD-exposed INS1 cells, but this was unremarkable compared with the ~440-fold increase in HepG2 cells (Fig. 2e). Moreover, changes in gene expression did not result in measureable increases in enzyme activity in INS1 cells or any other pancreatic endocrine cell lines tested (Fig. 2f).

### Functional CYP1A1 enzymes are strongly induced in human islets

**Fig. 3:**
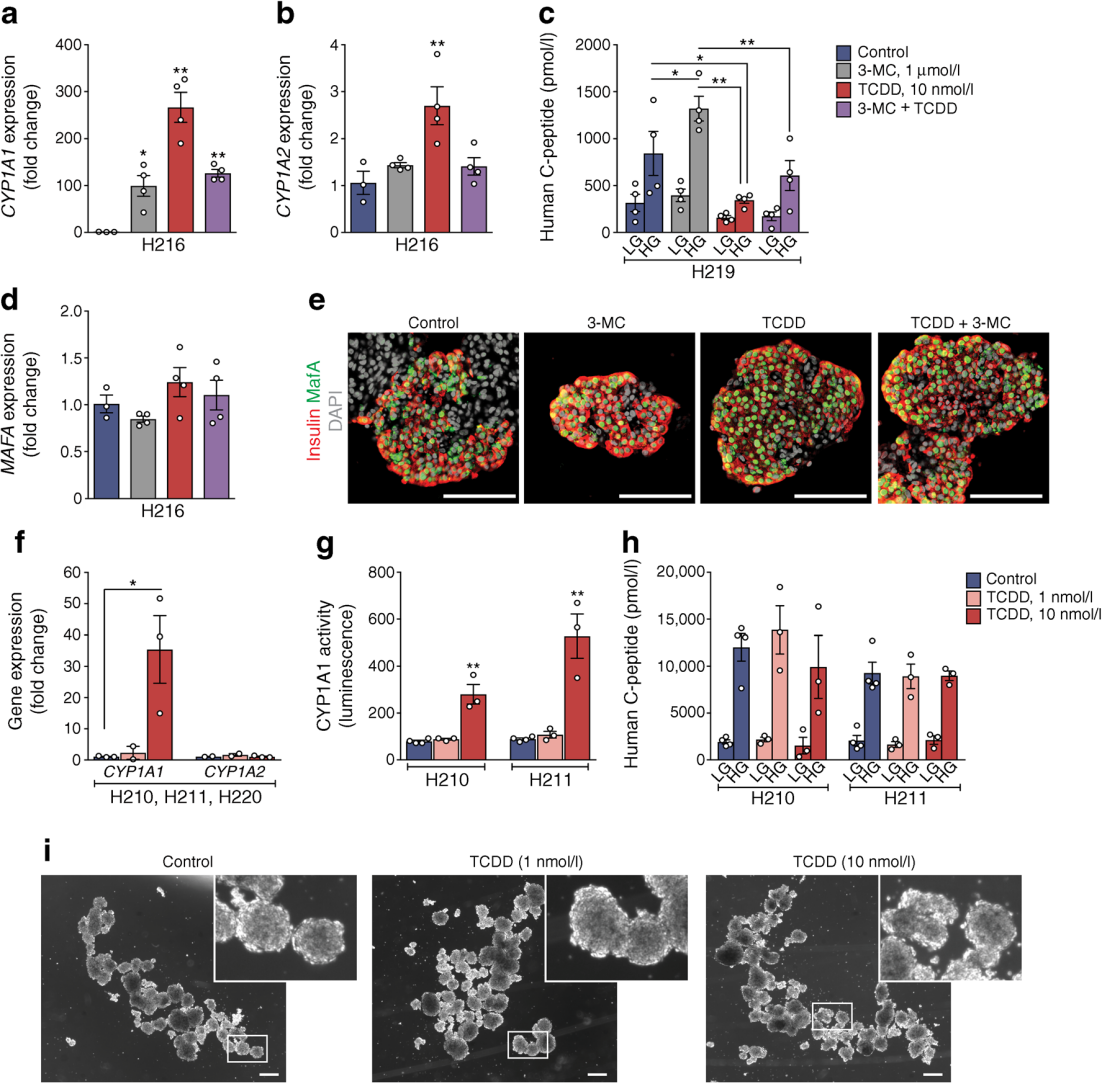
Functional CYP1A1 enzymes are induced in human islets following xenobiotic exposure in vitro*.* Human islets were treated with vehicle, or 3-MC (1 μmol/l) ± TCDD (1 or 10 nmol/l), for 48 h in vitro. (**a**, **b**, **d**) Gene expression of *CYP1A1*, *CYP1A2* and *MAFA*, expressed as the fold change relative to control. *p < 0.05, **p < 0.01 vs control (one-way ANOVA with Dunnet’s post hoc test). (**c**) Human C-peptide secretion after 1 h in LG (2.9 mmol/l) and HG (16.7 mmol/l) buffer. *p < 0.05, **p < 0.01 (two-way repeated measures ANOVA with Tukey’s post hoc test). (**e**) Representative images of human islets with immunofluorescence staining for insulin (red), MafA (green) and DAPI (grey). Scale bars represent 100 μm. (**f**) Gene expression of *CYP1A1* and *CYP1A2*, expressed as fold change relative to control. *p < 0.05 vs control (one-way ANOVA with Dunnet’s post hoc test). (**g**) CYP1A1 enzyme activity. **p < 0.01 vs control (one-way ANOVA with Dunnet’s post hoc test). (**h**) Human C-peptide secretion after 1 h in LG and HG buffer. (**i**) Representative brightfield images showing gross morphology of human islets after 48 h in vitro treatments. Scale bars represent 100 pixels. All data are presented as mean ± SEM. Individual data points represent technical replicates from a single organ donor (**a**, **b**, **d**: H216; **c**: H219; **g**, **h**: H210, H211), except for (**f**) where data points represent biological replicates from three organ donors (H210, H211, H220)

Since immortalised cell lines are not always an appropriate model, we next tested our hypothesis in primary human islets. Remarkably, both 3-MC and TCDD potently induced *CYP1A1* expression in human islets (~100-fold and ~270-fold, respectively; Fig. 3a), while *CYP1A2* was only modestly induced ~2.7-fold by TCDD (Fig. 3b). Interestingly, glucose-induced C-peptide secretion was increased by 3-MC, but inhibited by TCDD (Fig. 3c). Neither gene nor protein expression of MafA*,* a critical beta cell transcription factor, was affected by chemical exposure (Fig. 3d, e).

In three additional organ donors, *CYP1A1*, but not *CYP1A2*, was significantly upregulated by 10 nmol/l TCDD (Fig. 3f)*.* Importantly, this was associated with a significant increase in CYP1A1 enzyme activity (Fig. 3g), demonstrating that human islets harbour functional xenobiotic metabolism enzymes. In these donors, C-peptide secretion was not impacted by TCDD exposure (Fig. 3h). Islets appeared to be generally healthy, confirming that these doses of TCDD were not cytotoxic (Fig. 3i).

**Fig. 4:**
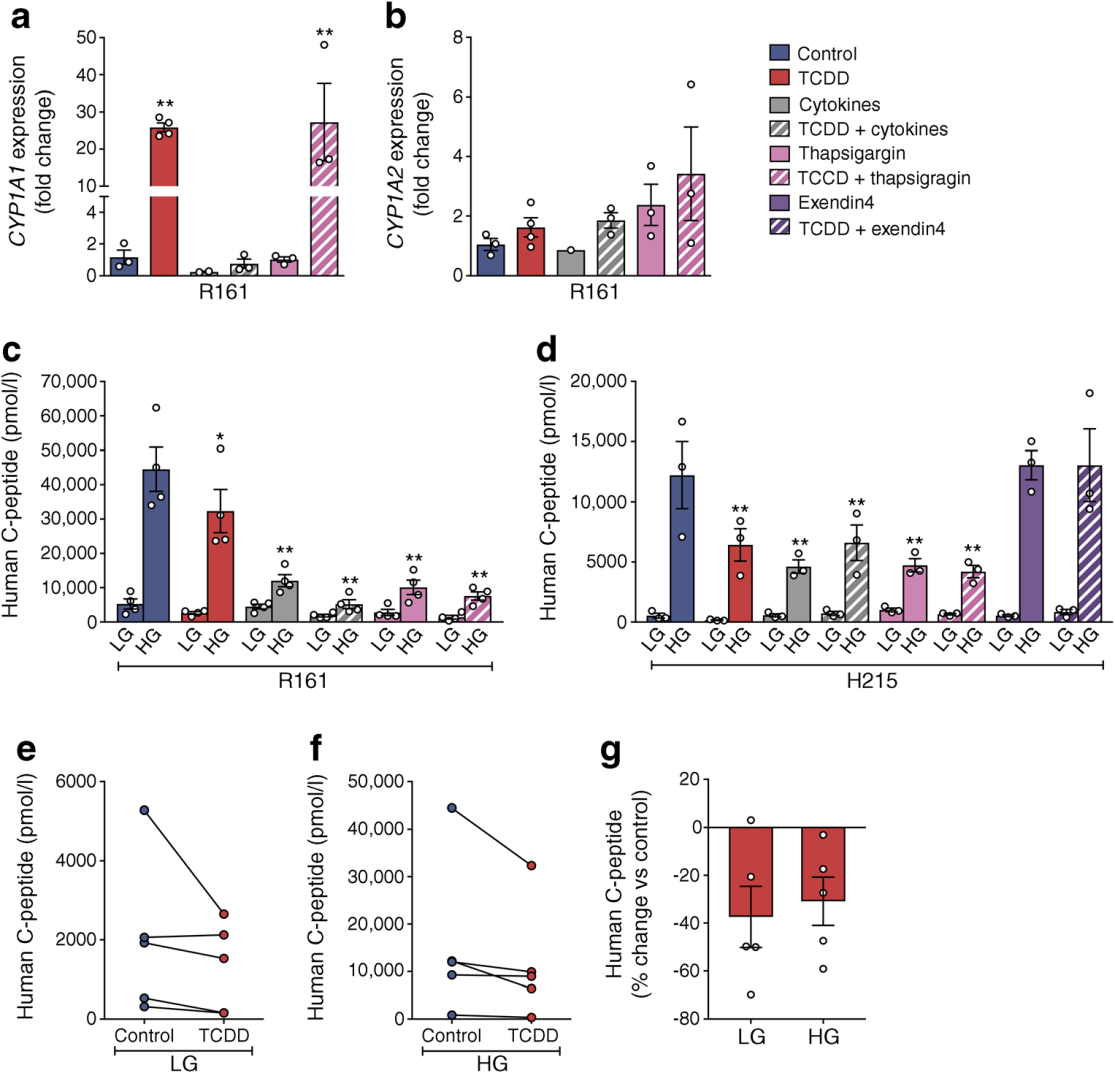
Cotreatment with cytokines, but not thapsigargin, interferes with *CYP1A1* induction in human islets. Human islets were treated with media containing DMSO (control), 10 nmol/l TCDD alone, cytokines (1000 U/ml IFNγ, 50 ng/ml TNF-α, 10 ng/ml IL1β) ± 10 nmol/l TCDD, 1 μmol/l thapsigargin ± 10 nmol/l TCDD, or 10 nmol/l exendin-4 ± 10 nmol/l TCDD for 48 h in vitro. (**a**, **b**) Gene expression of *CYP1A1* (**a**) and *CYP1A2* (**b**), expressed as fold change relative to control. **p < 0.01 vs control (one-way ANOVA with Dunnet’s post hoc test). (**c**, **d**) Human C-peptide after a 1 h incubation in LG (2.9 mmol/l) and HG (16.7 mmol/l) buffer. *p < 0.05, **p < 0.01 vs control (two-way repeated measures ANOVA with Dunnet’s post hoc test). (**e**–**g**) Compiled human C-peptide secretion data from five different organ donors, expressed as the mean human C-peptide concentration following LG (**e**) and HG (**f**) conditions, as well as the percentage change for TCDD-exposed islets relative to their respective control islets (**g**). A Wilcoxon matched-pairs signed rank test was performed to compare human C-peptide secretion for each organ donor with and without TCDD exposure (LG: *p* = 0.125, HG: *p* = 0.063). All data are presented as mean ± SEM. Individual data points represent technical replicates from a single organ donor (**a**–**c**: R161; **d**: H215), except for (**e**–**g**) where data points represent biological replicates from five different organ donors (H210, H211, H215, H219 and R161)

To determine whether other stressors induce *CYP1A* expression or interact with TCDD, human islets were cotreated with cytokines (to mimic inflammatory stress) or thapsigargin (to mimic endoplasmic reticulum stress). TCDD induced *CYP1A1* ~26-fold with or without thapsigargin (Fig. 4a). However, cotreatment with cytokines completely prevented *CYP1A1* induction by TCDD (Fig. 4a). *CYP1A2* gene expression was unchanged by any combination of stressors (Fig. 4b). TCDD significantly suppressed glucose-induced C-peptide secretion in two organ donors (Fig. 4c, d), and this effect was prevented by cotreatment with exendin-4 (Fig. 4d). Exposure to cytokines or thapsigargin also suppressed C-peptide secretion, irrespective of cotreatment with TCDD (Fig. 4c, d).

Given the variable insulin secretory responses from different organ donors (Figs. 3c, h, 4c, d), we compiled these results in Fig. 4e–g. Despite the biological variability in C-peptide production among donors, a consistent pattern of decreased C-peptide secretion was observed for each organ donor following TCDD treatment, particularly under HG conditions (*p* = 0.063; Fig. 4f). The change in glucose-induced C-peptide secretion by TCDD-treated (vs control) human islets ranged from −3% to −60% among the five donors (Fig. 4g).

### TCDD causes long-term CYP1A1 induction in mouse islets in vivo

**Fig. 5:**
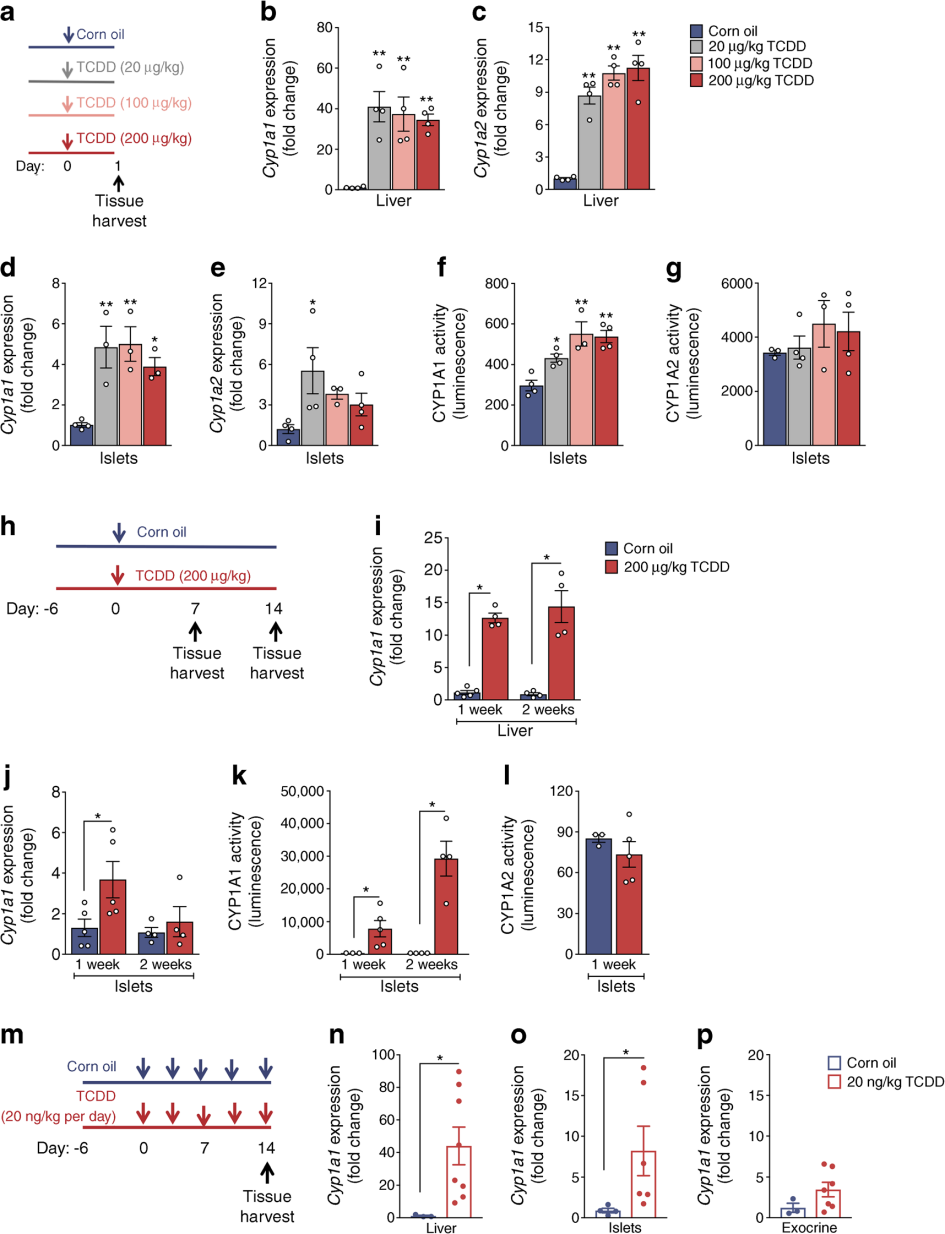
CYP1A1 gene expression and enzyme activity are induced in mouse islets in vivo*.* (**a**–**g**) Male C57Bl/6 mice were injected i.p. with either corn oil (vehicle) or TCDD (20 μg/kg, 100 μg/kg or 200 μg/kg) and euthanised 24 h later to harvest liver and isolate islets (schematic timeline shown in [**a**]). (**b**, **d**) *Cyp1a1* and (**c**, **e**) *Cyp1a2* gene expression expressed as fold change relative to controls in liver (**b**, **c**) and isolated islets (**d**, **e**). (**f**) CYP1A1 and (**g**) CYP1A2 enzyme activity was measured in islets at 24 h. (**h**–**l**) Male C57Bl/6 mice were injected i.p. with either corn oil or TCDD (200 μg/kg) and euthanised either 7 or 14 days later (schematic timeline shown in [**h**]). (**i**, **j**) *Cyp1a1* gene expression was measured in liver (**i**) and isolated islets (**j**) at 1 and 2 weeks post injection (expressed as fold change relative to control). (**k**) CYP1A1 and (**l**) CYP1A2 enzyme activity in isolated islets at 1 and 2 weeks post injection. (**m**–**p**) Male C57Bl/6 mice were injected i.p. with either corn oil or TCDD (20 ng/kg per day) twice per week and euthanised 14 days later (schematic timeline shown in [**m**]). (**n**–**p**) *Cyp1a1* gene expression was measured in liver (**n**), isolated islets (**o**) and pancreatic exocrine tissue (**p**) on day 14 (expressed as fold change relative to control). All data are presented as mean ± SEM. Individual data points represent biological replicates from different mice. (**b**–**g**) *p < 0.05, **p < 0.01 vs control, two-way repeated measures ANOVA with Dunnet’s post hoc test. (**i**–**l**, **n**–**p**) *p < 0.05, **p < 0.01 vs control, unpaired two-tailed *t* test

Our next question was whether functional CYP1A enzymes could be induced in islets following systemic TCDD exposure in vivo rather than direct exposure ex vivo*.* Mice were injected with a single high dose of TCDD (20, 100 or 200 μg/kg) or corn oil, and tissues harvested 24 h later (Fig. 5a). As expected, *Cyp1a1* and *Cyp1a2* were induced ~40-fold and ~10-fold, respectively, in the liver following TCDD exposure (Fig. 5b, c). In islets, *Cyp1a1* was induced ~5-fold by TCDD (all doses; Fig. 5d). *Cyp1a2* was only induced by 20 μg/kg TCDD (Fig. 5e). Most importantly, a dose-dependent increase in CYP1A1 enzyme activity (but not CYP1A2) was detected in islets 24 h post TCDD (Fig. 5f, g).

To assess the longevity of CYP1A1 activation, we followed a second cohort of mice for 2 weeks after a high-dose TCDD injection (200 μg/kg; Fig. 5h). *Cyp1a1* was induced ~12-fold in TCDD-exposed liver at 1 and 2 weeks post injection (Fig. 5i), compared with the ~35-fold increase after 24 h (Fig. 5b). In islets, *Cyp1a1* was elevated ~4-fold at 1 week post TCDD, but unchanged at 2 weeks (Fig. 5j). However, while *Cyp1a1* gene levels were declining in islets, CYP1A1 enzyme activity increased dramatically (Fig. 5k). Whereas only a 1.9-fold increase in CYP1A1 activity was detected in TCDD-exposed islets at 24 h (Fig. 5f), CYP1A1 activity increased ~40-fold and ~80-fold in islets at 1 and 2 weeks, respectively, after the single TCDD injection (Fig. 5k). Islet CYP1A2 activity was unchanged (Fig. 5l).

We performed immunofluorescence staining on pancreas sections from TCDD-injected mice to localise CYP1A1 expression within islets, but unfortunately the CYP1A1 antibodies we tested were unreliable (ESM Table 1). Immunofluorescence was either undetectable in TCDD-exposed liver and pancreas or the same pattern of immunoreactivity was observed in both wild-type (WT) and *Cyp1a1*/*1a2* knockout (KO) mice, indicating non-specific immunoreactivity (representative examples shown in ESM Fig. 1). Therefore, we can conclude that CYP1A1 is upregulated in whole islets, but cannot comment on whether it is induced in beta cells specifically.

Finally, we investigated whether a more physiologically relevant TCDD dosing protocol would induce CYP1A1 enzymes in islets. Mice were injected two times per week with 20 ng/kg TCDD (Fig. 5m), which is 10,000 times lower than the single high-dose model (Fig. 5a–l). After 2 weeks *Cyp1a1* levels were upregulated ~40-fold in liver (Fig. 5n) and ~8-fold in islets (Fig. 5o). This was comparable to the degree of *Cyp1a1* induction 24 h after a single high-dose TCDD injection (Fig. 5b, d). *Cyp1a1* levels did not change in TCDD-exposed pancreatic exocrine tissue (Fig. 5p).

### TCDD exposure suppressed glucose-stimulated insulin secretion

**Fig. 6:**
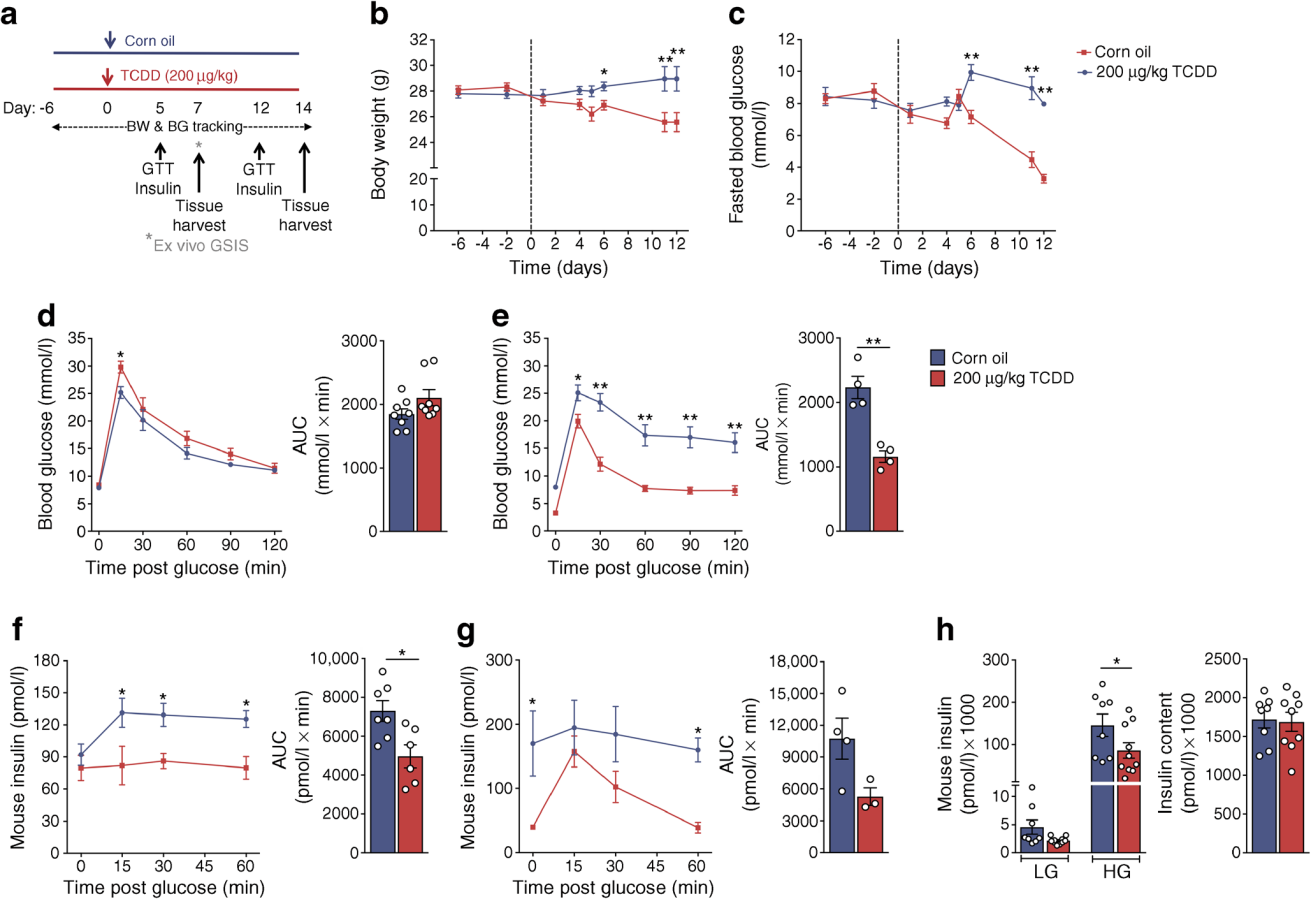
A single high-dose TCDD injection leads to suppressed plasma insulin levels in vivo. Male C57Bl/6 mice were injected i.p. with either corn oil (vehicle) or TCDD (200 μg/kg) and euthanised either 7 or 14 days later (schematic timeline shown in [**a**]). BG, blood glucose; BW, body weight. (**b**) Body weight and (**c**) blood glucose were measured after a 4 h morning fast throughout the study. (**d, e**) Blood glucose and (**f**, **g**) plasma insulin were measured during a GTT on days 5 (**d**, **f**) and 12 (**e**, **g**). (**h**) On day 7 islets were isolated and glucose-stimulated insulin secretion (GSIS) was measured ex vivo after 1 h in LG (2.9 mmol/l) and 1 h in HG (16.7 mmol/l), followed by total insulin content measurement. All data are presented as mean ± SEM. Individual data points represent biological replicates from different mice. *p < 0.05, **p < 0.01 vs control; (**b**, **c**) two-way ANOVA with Šídák multiple comparisons test, (**d**–**h**) two-way repeated measures ANOVA with Šídák multiple comparisons test, (**d**–**g**) unpaired two-tailed *t* test for AUC

Activation of CYP1A1 enzymes in islets strongly suggests that islets are directly exposed to TCDD in vivo. Therefore, we investigated the impact of TCDD on beta cell function and survival rate*.* We first characterised the mice exposed to a single high dose of TCDD (Fig. 6a) in which islet CYP1A1 enzyme activity was dramatically upregulated at 1 and 2 weeks (Fig. 5k). These mice can only be maintained for ~2 weeks, as their health begins to decline thereafter [47]. Beginning at 6 days post TCDD, we observed modest but significant body weight loss (~5%; Fig. 6b) and a drastic decline in fasting blood glucose levels (Fig. 6c). However, on day 5 TCDD-exposed mice had slightly higher blood glucose levels 15 min after a glucose challenge with no overall change in glucose tolerance (Fig. 6d). By day 12, TCDD-exposed mice were profoundly hypoglycaemic during the GTT (Fig. 6e). Plasma insulin levels were signficantly lower in TCDD-exposed mice during the GTT on both days (Fig. 6f, g).

In contrast to the single high-dose TCDD, glucose homeostasis was largely unaffected by multiple low-dose TCDD exposures for 25 days (ESM Fig. 2). Fasting glucose levels were modestly decreased in TCDD-exposed mice (ESM Fig. 2c), but there was no difference in glucose tolerance (ESM Fig. 2e), body weight (ESM Fig. 2b), insulin sensitivity (ESM Fig. 2d) or plasma insulin levels during the GTT (ESM Fig. 2f).

**Fig. 7:**
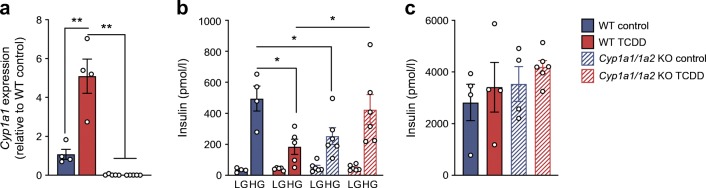
TCDD suppressed glucose-stimulated insulin secretion in male WT islets but not *Cyp1a1*/*1a2* KO islets. Islets were isolated from male WT mice and mice with a global KO of *Cyp1a1* and *Cyp1a2* (*Cyp1a1*/*1a2* KO) and treated ex vivo for 48 h with 10 nmol/l TCDD*.* (**a**) *Cyp1a1* gene expression was expressed relative to WT control levels. (**b**) Insulin secretion was measured ex vivo after 1 h in LG (2.9 mmol/l) and 1 h in HG (16.7 mmol/l), followed by (**c**) total insulin content in islets. All data are presented as mean ± SEM. Individual data points represent biological replicates from different mice. *p < 0.05, **p < 0.01; (**a**) one-way ANOVA with Tukey’s multiple comparisons test, (**b**) two-way repeated measures ANOVA with Tukey’s multiple comparisons test

To better understand whether the low plasma insulin levels following high-dose TCDD (Fig. 6f, g) were caused by impaired beta cell function vs adaptation to other effects of TCDD (e.g. hepatic toxicity, increased insulin clearance), islets were isolated on day 7. Interestingly, glucose-stimulated insulin secretion was suppressed in islets from TCDD-injected mice compared with controls ex vivo (Fig. 6h). Next, islets were isolated from *Cyp1a1*/*Cyp1a2* KO or WT mice and treated with TCDD ex vivo to determine whether induction of CYP1A1 in islets might be involved in suppressing insulin secretion. As expected, *Cyp1a1* was induced by TCDD in WT islets and non-detectable in KO islets, irrespective of treatment (Fig. 7a). Consistent with our findings in vivo (Fig. 6) and in human islets (Figs. 3c, 4c–g), TCDD suppressed glucose-stimulated insulin secretion in WT islets (Fig. 7b). In contrast, TCDD did not suppress insulin secretion in *Cyp1a1*/*1a2* KO islets, although control KO islets had unexpectedly lower insulin secretion than WT islets (Fig. 7b), suggesting a role for basal CYP1A1/CYP1A2 in regulating beta cell function. Total insulin content was not affected by genotype or TCDD (Fig. 7c).

### TCDD causes beta cell death and suppression of anti-apoptotic genes in islets

**Fig. 8:**
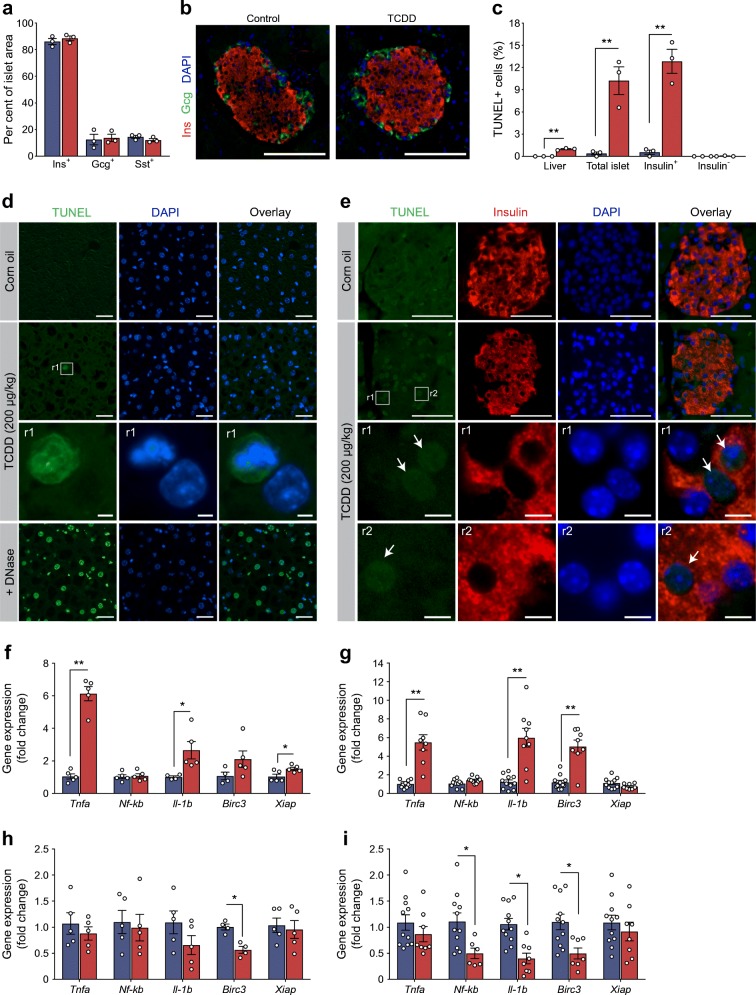
TCDD causes beta cell death in vivo*.* Male C57Bl/6 mice were injected i.p. with either corn oil (vehicle) or TCDD (200 μg/kg) and euthanised either 7 or 14 days later. (**a**) The percentage of total islet area that was immunoreactive for insulin (Ins^+^), glucagon (Gcg^+^) and somatostatin (Sst^+^) was quantified in pancreas sections collected at 7 days post injection. (**b**) Representative images of islets with insulin (red) and glucagon (green) immunoreactivity are shown. Scale bars represent 100 μm. (**c**) The percentages of liver cells and islet cells that coexpressed TUNEL were quantified in tissue sections at 1 week post injection. Within islets, TUNEL^+^ cells were further delineated into insulin^+^ and insulin^−^ cells. (**d**, **e**) Representative images of liver (**d**) and pancreas (**e**) showing TUNEL (green), insulin (red) and DAPI (blue) as individual channels and overlay images. Inset regions within the white boxes (labelled r1 and r2) are shown magnified below. Scale bars represent 50 μm, except for inset regions, which are 5 μm. Images from liver sections treated with DNase as a postive control are also provided (**d**). Arrows in (**e**) indicate TUNEL^+^ cells. (**f**–**i**) Expression of genes involved in inflammation and apoptosis was measured in liver (**f**, **g**) and isolated islets (**h**, **i**) on day 7 (**f**, **h**) and day 14 (**g**, **i**). Gene levels are expressed as fold change relative to control. All data are presented as mean ± SEM and individual data points represent biological replicates from different mice. *p < 0.05, **p < 0.01 vs control, unpaired two-tailed *t* test

Reduced plasma insulin can also be caused by beta cell loss, so we next assessed pancreas histology. Surprisingly, pancreas morphology appeared relatively normal by H&E staining, unlike the liver, which showed obvious signs of inflammation and cell damage (ESM Fig. 3). Likewise, there was no change in endocrine cell composition within islets (percentage insulin^+^, glucagon^+^ and somatostatin^+^ area) at 7 days post TCDD (Fig. 8a, b). There was, however, a remarkably high percentage of TUNEL^+^ islet cells, nearly all of which were insulin^+^ rather than insulin^−^ (Fig. 8c, e). On average, ~13% of insulin^+^ cells were TUNEL^+^ (Fig. 8c, e), compared with only ~1% TUNEL^+^ liver cells in the same mice (Fig. 8c, d). At both 7 and 14 days post TCDD, the liver had significant induction of proinflammatory cytokines (*Tnfa*, *Il-1b*), as well as inhibitors of apoptosis *Birc3* and *Xiap* (Fig. 8f, g)*.* In stark constrast, TCDD significantly suppressed *Birc3* expression in islets on days 7 and 14 (Fig. 8h, i), as well as *Il-1b* and *Nf-kb* on day 14 (Fig. 8i).

## Discussion

Our study demonstrates that xenobiotic metabolising CYP enzymes are activated within pancreatic islets following pollutant exposure. Specifically, CYP1A1 gene expression and enzyme activity were induced in human and mouse islets following direct TCDD exposure in vitro, and in mouse islets following systemic TCDD exposure in vivo*.* The enzyme activity assay measures CYP1A1-mediated metabolism of a proluciferin substrate into d-luciferin, indicating that the upregulated *CYP1A1* gene is indeed translated into functional enzymes capable of producing metabolites. To our knowledge, this is the first evidence of functional CYP enzymes in the endocrine pancreas. Remarkably, the activity level of CYP1A1 enzymes in islets increased for at least 2 weeks after a single high-dose TCDD injection, even though *Cyp1a1* gene expression in islets had returned to baseline. Moreover, this pathway was activated in islets by both a supraphysiological dose of TCDD in vivo and a biologically relevant, multiple low-dose exposure protocol.

These findings have several important implications. First and foremost, local activation of CYP1A1 in islets provides strong evidence that environmental pollutants reach pancreatic endocrine cells in vivo at sufficient levels to directly alter cell signalling. Given the highly specialised nature of endocrine cells, the signalling pathways activated by dioxin in islets will likely differ from classical target tissues. This was apparent in our analysis of inflammatory signalling pathways in liver vs islets from TCDD-exposed mice. In liver, TCDD profoundly upregulated proinflammatory cytokines, along with anti-apoptotic genes *Birc3 and Xiap*, likely as a protective response. However, TCDD significantly downregulated *Birc3*, an inhibitor of beta cell apoptosis [48], at 1 and 2 weeks in islets and did not induce cytokines. *Birc3* activates *Nf-kb* [48], so the subsequent decrease in *Nf-kb* and *ll-1b* at 2 weeks is consistent with TCDD suppressing *Birc3* in islets. Nf-κb is also reportedly anti-apoptotic in beta cells [49], whereas Il-1b can be either pro-apoptotic or protective depending on timing and concentration [50–52]. The absence of an inflammatory and anti-apoptotic response in islets was associated with a dramatic increase in beta cell apoptosis, compared with only a modest increase in liver cell death in TCDD-exposed mice. Although a detailed mechanism remains to be elucidated, our data show that while TCDD induces CYP1A1 in liver and islets, the consequences of chemical exposure are profoundly different at both molecular and cellular levels.

Our study also shows that a single transient chemical exposure results in long-term activation of CYP1A1 enzymes within islets. The relatively slow decline of TCDD levels in pancreas [37], combined with the long-term induction of CYP1A1 activity in islets, suggests that pancreatic cells could be a ‘sink’ for long-term storage of lipophilic chemicals. Furthermore, the induction of *Cyp1a1* in islets, but not exocrine tissue, suggests that whole pancreas measurements of TCDD may underestimate islet exposure. We speculate that the dense intra-islet vascular network, designed to ensure rapid delivery of oxygen and nutrients to endocrine cells, also promotes delivery of xenobiotics to islets. Furthermore, the degree of CYP1A1 activation in islets was surprisingly high (~5–8-fold lower than liver) considering the relatively small proportion of TCDD that reaches the pancreas. This supports both our hypothesis that islets are exposed to disproportionately high levels of TCDD and conclusions by Diliberto and colleagues [37] that some tissues require lower levels of TCDD to ellicit a response. Whether islets are exposed to more TCDD than predicted by biodistribution studies, or are highly sensitive to TCDD, our data conclusively show that CYP1A1 enzymes are activated locally following environmental chemical exposure.

Neither thapsigargin nor cytokines activated AhR signalling in human islets, but TCDD was surprisingly unable to induce *CYP1A1* in the presence of proinflammatory cytokines. This could have important implications for how individuals with islet inflammation, including those with type 2 diabetes [53–56], respond to environmental chemicals that activate AhR signalling. For instance, if activation of CYP1A1 in islets is an important defence pathway, then islets with increased inflammation may be more sensitive to injury caused by environmental chemicals. On the other hand, if upregulation of CYP1A1 in islets is detrimental (for example, due to the formation of reactive metabolites), then islets in a proinflammatory state might be somewhat protected from the toxic effects of TCDD or other AhR ligands. Indeed, our data suggest that CYP1A enzymes may play a non-conventional role in islets. Direct TCDD exposure suppressed glucose-stimulated insulin secretion in WT mouse islets*,* but not in *Cyp1a1*/*1a2* KO islets, suggesting that CYP1A1 induction might inhibit insulin secretion. However, *Cyp1a1*/*1a2* KO control islets also showed an unexpected decrease in insulin secretion compared with WT controls, pointing to a possible role for basal CYP1A1 in regulating beta cell function. More research is required to understand the role of CYP1A1 enzymes in islet physiology and possible interactions with proinflammatory cytokines.

The single high dose of TCDD used in these studies (200 μg/kg) was selected to study CYP1A1 activation in islets and was not ideal for assessing the impact of TCDD on beta cell function*.* After 1 week, TCDD-exposed mice displayed weight loss and hypoglycaemia, likely resulting from hepatotoxicity. This is consistent with previous reports [47, 57, 58] and confounds our interpretation of potential direct effects of TCDD on islets. However, on day 5, when blood glucose levels were still within a healthy range, TCDD-exposed mice displayed reduced plasma insulin levels during a GTT*.* Suppressed insulin secretion was also detected in isolated islets ex vivo on day 7 post TCDD, which is consistent with previous findings in isolated islets 24 h after a high dose of TCDD in vivo [30, 31]. Furthermore, when human islets were exposed directly to TCDD*,* three of five donors had significantly lower C-peptide secretion and, on average, all donor islets secreted less C-peptide if they had been exposed to TCDD vs DMSO. Mouse islets also showed a similar response to direct TCDD treatment ex vivo*.* Together, these data suggest that TCDD directly affects islet function, possibly due to direct impairment of insulin secretion by beta cells or via indirect paracrine mechanisms (i.e. secretion of counterregulatory hormones). The impact of TCDD on islet physiology should be further assessed in longer-term studies at more physiologically relevant concentrations.

Exposure to dioxin-like pollutants is consistently associated with increased type 2 diabetes incidence and beta cell dysfunction in humans [24, 25, 27, 28], yet the pancreas has been largely overlooked as a potential target tissue. We show that systemic TCDD administration upregulates CYP1A1 enzymes in islets, indicating that the endocrine pancreas is directly exposed to TCDD in vivo*.* We expect that other dioxin-like pollutants will activate the AhR pathway in islets in a similar manner, although presumably with less potency than TCDD. The role of CYP1A1 in islets generally and beta cells specificially remains to be determined, but, at minimum, this enzyme serves as a useful biomarker for direct exposure of the endocrine pancreas to POPs in vivo*.*

## Electronic supplementary material

ESM (PDF 21724 kb)

## Data Availability

The datasets generated during and/or analysed during the current study are available from the corresponding author on reasonable request.

## Abbreviations

3-MC: 3-Methylcholanthrene
AhR: Aryl hydrocarbon receptor
CYP: Cytochrome P450
CYP1A1: Cytochrome P450 1A1
CYP1A2: Cytochrome P450 1A2
DMEM-HG: High-glucose DMEM
HBSS: Hanks’ balanced salt solution
HG: High glucose
HIER: Heat-induced epitope retrieval
KO: Knockout
KRBB: Krebs–Ringer bicarbonate buffer
LG: Low glucose
MafA: MAF bZIP transcription factor A
PFA: Paraformaldehyde
POPs: Persistent organic pollutants
qPCR: Quantitative real-time PCR
TCDD: 2,3,7,8-Tetrachlorodibenzo*-p-*dioxin
WT: Wild type
