# Supplementary material for: Functional cytochrome P450 1A enzymes are induced in mouse and human islets following pollutant exposure
Source: Diabetologia. 2019 Nov 27;63(1):162–78. doi: 10.1007/s00125-019-05035-0 (PMC6890627; doi:10.1007/s00125-019-05035-0)
Supplement: Supplementary file 1 — (PDF 21724 kb) [file 125_2019_5035_MOESM1_ESM.pdf]

ESM Table 1 Primer sequences for qPCR

**Target**	**Species**	**Forward Sequence (5'-3')**	**Reverse Sequence (5'-3')**
PPIA	Human/ Mouse	AGCTCTGAGCACTGGAGAGA	GCCAGGACCTGTATGCTTTA
Hprt	Mouse	GCT GAC CTG CTG GAT TAC AT	TTG GGG CTG TAC TGC TTA AC
HPRT	Human	TGTTGTAGGATATGCCCTTGACTAT	GCGATGTCAATAGGACTCCAGA
Cyp1a1	Mouse	ATCACAGACAGCCTCATTGAGC	AGATAGCAGTTGTGACTGTGTC
CYP1A1	Human	GAA CAA ACA GGG CTG CCT TCT	GAG ACC AAT AGA TAA TTG AAA TAC CCC
Cyp1a2	Mouse	CAAGAGGTTTAAGACCTTCAATGATAAC	AAAGATGTCATTGACAATGTTGACAAT
CYP1A2	Human	GTA TTT TTA GTA GAG ACG GGT TTC ACC AT	GCA GGG TTT CTT TTA GGG GTG ATG
Tnfa	Mouse	AGT CCG GGC AGG TCT ACT TT	ATG AAC ACC CAT TCC CTT CA
Nf-kb	Mouse	CTC AGG AGC AGA AGT CTG GG	GCC GCT ATA TGC AGA GGT GT
Il-1b	Mouse	GCC ACC TTT TGA CAG TGA TGA G	AGC TTC TCC ACA GCC ACA AT
Birc3	Mouse	CCC GGA GAT CAG AGG TCA TTG	GAA AGG CGC TGT CTT GAA CC
Xiap	Mouse	TCG GGT CAG CCT CCT TAA AC	TGG TGT CTG CAA GTA CAA AAG T

ESM Table 2 CYP1A1 antibodies tested in pancreas and liver tissue from TCDD-exposed mice

**Antigen**	**Species**	**Company**	**Catalogue #**	**Dilutions tested**
CYP1A1/1A2	Mouse	AbCam	ab111868	1:100, 1:1000
CYP1A1	Rabbit	AbCam	ab79819	1:100, 1:1000
CYP1A1	Rabbit	Invitrogen	PA5-15213	1:50, 1:25, 1:10
CYP1A1	Mouse	SantaCruz	sc-25304	1:50
CYP1A1	Mouse	Thermo Fisher	MA5-17063	1:200
CYP1A1	Rabbit	GeneTex	75868-464	1:100

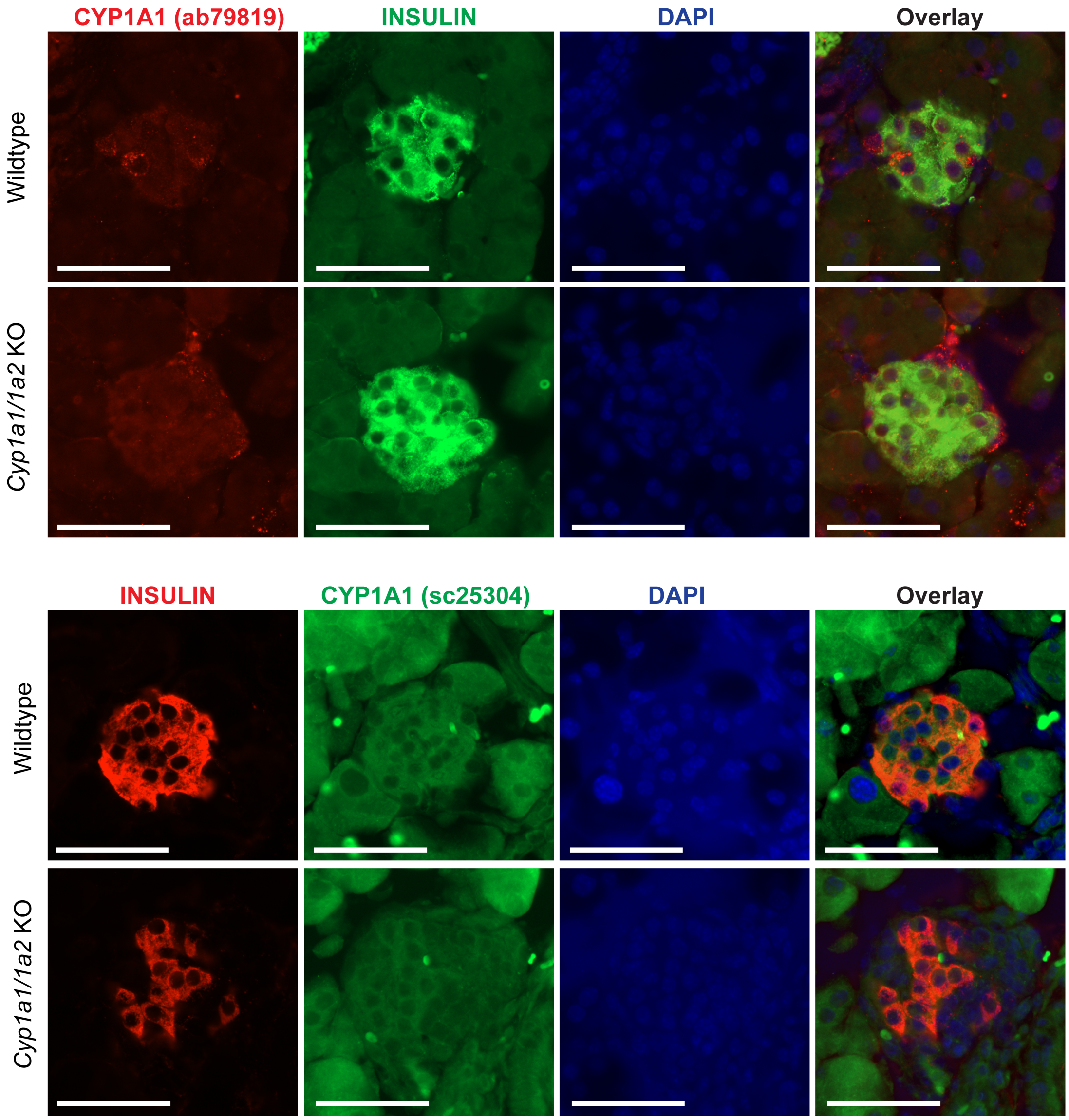

ESM Fig. 1 Immunofluorescence staining of pancreas from wildtype mice and *Cyp1a1/1a2* double knockout mice. Representative images from immunostaining with antibodies for insulin and CYP1A1 in pancreas sections from wildtype mice and *Cyp1a1/1a2* double knockout mice. Individual channels are shown along with overlay images. Scale bars represent 50 µm.

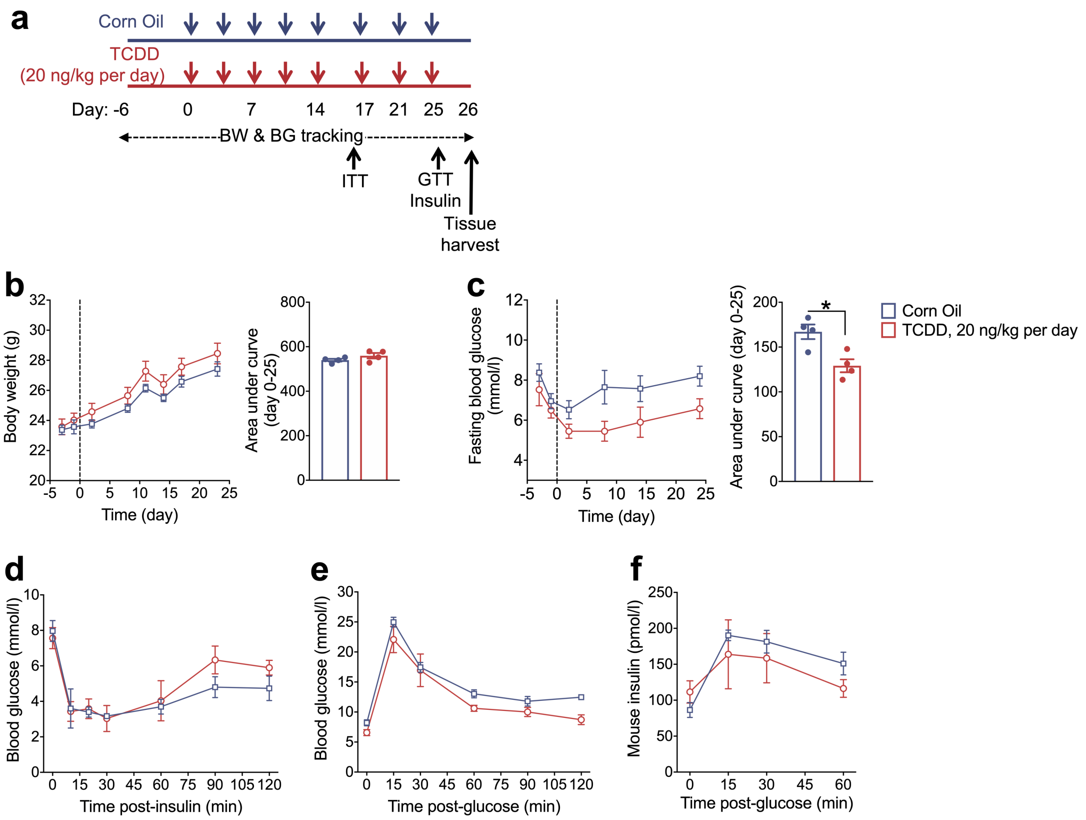

ESM Fig. 2 Multiple low-dose TCDD injections did not impact glucose homeostasis *in vivo.* Male C57Bl/6 mice were injected i.p. with either corn oil (vehicle) or TCDD (20 ng/kg per day) twice per week and euthanized 26 days later (schematic timeline shown in **[a]**). **(b)** Body weight (BW) and **(c)** blood glucose (BG) was measured after a 4 h morning fast throughout the study. **(b-c)** Area under the curve is shown for BW and BG measurements between day 0-25. *p<0.05, unpaired two-tailed *t* test. **(d)** An insulin tolerance test (ITT) was performed on day 17. **(e)** Blood glucose and **(f)** plasma insulin levels were measured during a glucose tolerance test (GTT) on day 25. All data are presented as mean±SEM. Individual data points represent biological replicates from different mice.

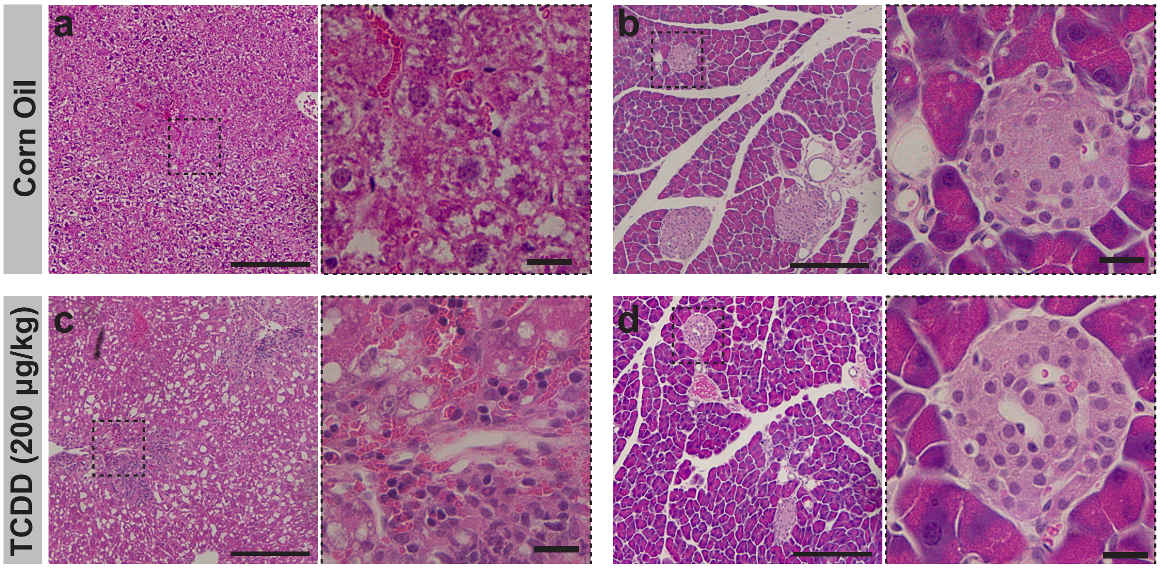

ESM Fig. 3 Histology of liver and pancreas from control and TCDD-exposed mice after 7 days*.* Representative images of hematoxylin & eosin (H&E)-stained sections of liver **(a,c)** and pancreas **(b,d)** tissues from mice injected with corn oil **(a,b)** or 200 µg/kg TCDD **(c,d).** Inset regions within the dotted black boxes are shown magnified to the right of each image. Scale bars represent 200 µm in low-magnification images and 20 µm in inset regions.

Checklist for reporting human islet preparations used in research

Adapted from Hart NJ, Powers AC (2018) Progress, challenges, and suggestions for using human islets to understand islet biology and human diabetes. Diabetologia https://doi.org/10.1007/s00125-018-4772-2

Islet preparation	1	2	3	4	5	6	7
**MANDATORY INFORMATION**							
Unique identifier	H216	H219	H210	H211	H220	R161	H215
Donor age (years)	27	58	18	17	55	71	18
Donor sex (M/F)	F	M	F	F	M	F	M
Donor BMI (kg/m^2)	22.9	28.4	20.1	22.9	28.4	19.7	
Donor HbA_{1c} or other measure of blood glucose control	Not available	Not available	Not available	Not available	Not available	6.3	Not available
Origin/source of islets[b]	Ike Barber Human Islet Transplant Laboratory	Ike Barber Human Islet Transplant Laboratory	Ike Barber Human Islet Transplant Laboratory	Ike Barber Human Islet Transplant Laboratory	Ike Barber Human Islet Transplant Laboratory	ADI IsletCore	Ike Barber Human Islet Transplant Laboratory
Islet isolation centre	Ike Barber Human Islet Transplant Laboratory	Ike Barber Human Islet Transplant Laboratory	Ike Barber Human Islet Transplant Laboratory	Ike Barber Human Islet Transplant Laboratory	Ike Barber Human Islet Transplant Laboratory	ADI IsletCore	Ike Barber Human Islet Transplant Laboratory
Donor history of diabetes?	No	No	No	No	No	No	No
**If Yes, complete the next two lines if this information is available**							
Diabetes duration (years)	N/A	N/A	N/A	N/A	N/A	N/A	N/A
Glucose-lowering therapy at time of death[c]							
**RECOMMENDED INFORMATION**							
Donor cause of death						NDD - Neurological	
Warm ischaemia time (h)							
Cold ischaemia time (h)						11.6	
Estimated purity (%)	60%	60%	90%	80%	70%	95%	90%
Estimated viability (%)							
Total culture time (h)[d]	40 h at 22C pre-shipping	8 h at 22C pre-shipping	48 h at 22C pre-shipping	24 h at 22C pre-shipping			36 h at 22C pre-shipping
Glucose-stimulated insulin secretion or other functional measurement[e]							
Handpicked to purity?	No	No	No	No	No	No	No
Additional notes							

[a]If you have used more than eight islet preparations, please complete additional forms as necessary
[b]For example, IIDP, ECIT, Alberta IsletCore
[c]Please specify the therapy/therapies
[d]Time of islet culture at the isolation centre, during shipment and at the receiving laboratory
[e]Please specify the test and the results
